# Charge screening and hydrophobicity drive progressive assembly and liquid–liquid phase separation of reflectin protein

**DOI:** 10.1016/j.jbc.2025.108277

**Published:** 2025-02-06

**Authors:** Reid Gordon, Robert Levenson, Brandon Malady, Yahya Al Sabeh, Alan Nguyen, Daniel E. Morse

**Affiliations:** Department of Molecular, Cellular, and Developmental Biology and the Institute for Collaborative Biotechnologies, University of California, Santa Barbara, California, USA

**Keywords:** biomaterials, biophotonics, cephalopods, fluorescence recovery after photobleaching (FRAP), fluorescence resonance energy transfer (FRET), electrostatics, iridescence, liquid–liquid phase separation, protein clusters, protein self-assembly, reflectins

## Abstract

The intrinsically disordered reflectin proteins drive tunable reflectivity for dynamic camouflage and communication in the recently evolved *Loliginidae* family of squid. Previous work revealed that reflectin A1 forms discrete assemblies whose size is precisely predicted by protein net charge density and charge screening by the local anion concentration. Using dynamic light scattering, FRET, and confocal microscopy, we show that these assemblies, of which 95 to 99% of bulk protein in solution is partitioned into, are dynamic intermediates to liquid protein-dense condensates formed by liquid–liquid phase separation (LLPS). Increasing salt concentration drives this progression by anionic screening of the cationic protein’s Coulombic repulsion, and by increasing the contribution of the hydrophobic effect which tips the balance between short-range attraction and long-range repulsion to drive protein assembly and ultimately LLPS. Measuring fluorescence recovery after photobleaching and droplet fusion dynamics, we demonstrate that reflectin diffusivity in condensates is tuned by protein net charge density. These results illuminate the physical processes governing reflectin A1 assembly and LLPS and demonstrate the potential for reflectin A1 condensate-based tunable biomaterials. They also compliment previous observations of liquid phase separation in the Bragg lamellae of activated iridocytes and suggest that LLPS behavior may serve a critical role in governing the tunable and reversible dehydration of the membrane-bounded Bragg lamellae and vesicles containing reflectin in biophotonically active cells.

The skin of cephalopods contains a complex optical system combining both pigmentary and structural coloration working in tandem (1, 2, 3). Iridocytes and leucophore cells produce structural color using subwavelength nanostructures (4). Unlike broad-band scattering leucophores, iridocytes reflect light in an angle- and wavelength-dependent manner *via* extensive and highly regular membrane invaginations, forming stacks of Bragg lamellae densely filled with the reflectin proteins (5, 6, 7, 8). Uniquely, the recently evolved *Loliginidae* squid possess reversibly and finely tunable leucophores and iridocytes. In the iridocytes, neuronally released acetylcholine binds muscarinic receptors, activating a signal transduction pathway (9) that results in the phosphorylation of reflectin proteins, neutralizing Coulombic repulsion of these cationic, intrinsically disordered proteins to drive their resulting folding and hierarchical assembly (5, 6, 10). It has been hypothesized that this assembly drives the cells’ colligative, osmotic dehydration (11), an effect augmented by steric dehydration and by the release of counterions from the folding and condensation of reflectins resulting in Gibbs-Donnan equilibration, all combining to drive a net efflux of water out from the Bragg lamellae upon activation and a net influx upon reversal (12). This reversible dehydration proportionally increases the refractive index of the Bragg lamellae and reduces their thickness and spacing, thus progressively tuning the wavelength and increasing the intensity of reflected light (5, 7, 8, 11, 12). Essentially the same mechanism controls the tunable dehydration and broad-band reflectivity of the reflectin-containing vesicles in the tunably white leucophores (13, 14).

Reflectin proteins are enriched in arginine, aromatic residues, and methionine while being devoid of lysine and aliphatic residues (6, 10, 15, 16). They are block copolymers of cationic linkers and the strongly conserved reflectin repeat motifs (RMs) and N-terminal sequence (16, 17); these motifs are methionine-rich and show little sequence similarity to nonreflectin proteins (Fig. S1) (16, 17). The abundance of RY and GRY sequences in reflectin A1 from *Doryteuthis opalesc*ens suggests that cation–pi interactions provide a major driving force in reflectin folding and assembly (18, 19). Similarly, the enrichment of methionine residues suggests that sulfur-pi bonds could contribute to interprotein and intraprotein interactions (18, 20), which is supported by solution NMR spectroscopy of a folded, truncated reflectin peptide (20). Increasing pH progressively deprotonates the abundant histidine residues in reflectin A1, reducing the number of positive charges and acting as an *in vitro* surrogate for the charge neutralization resulting from phosphorylation (11, 17). This reduction in protein net charge density (NCD) causes the reversible folding, as characterized by the formation of CD signatures of secondary structure and disappearance of the disordered signature, and proportional assembly of reflectin A1 into higher order multimers (11, 17). Altering reflectin A1 protein NCD by pH titration of histidine residues, genetic insertion of charged residues, and electrochemical titration reveals that the NCD of the cationic linker regions opposes assembly, and progressively reducing their Coulombic repulsion increases assembly size (17, 21, 22, 23). This remarkable *in vivo* and *in vitro* tunability of reflectin behavior has drawn much bioengineering interest for creating optically tunable biomaterials (24, 25, 26, 27, 28, 29).

Reflectin A1 exhibits several features consistent with proteins that undergo liquid–liquid phase separation (LLPS) (18, 19, 30, 31); it is an intrinsically disordered protein rich in arginine and tyrosine, and its sequence exhibits a pronounced regularity of charge patterning. LLPS of proteins into protein-dense and protein-dilute phases has been demonstrated to form membraneless organelles in living cells that often are regulated by posttranslational modifications such as phosphorylation (32, 33, 34, 35). These liquid-like condensates can act to sequester other proteins and RNA, organize and regulate signaling complexes, and buffer intercellular protein concentrations (36, 37, 38). Acetylcholine-activated reflectin phosphorylation drives reflectin LLPS *in vivo*, demonstrated by ultrastructural changes in the Bragg lamellae and leucocyte vesicles where discrete 10 to 50 nm diameter particles transition to a uniform, densely staining liquid (5, 13, 39). Isolated Bragg lamellae from activated iridocytes of the Loliginid squid *Lolliguncula brevis* demonstrated liquid properties such as deformability and surface wetting (39). Additionally, recent simulations based on short-angle X-ray scattering of reflectin A1 demonstrate a transition from oligomeric assemblies to LLPS (40).

Here, we demonstrate using FRET and dynamic light scattering (DLS) that reflectin A1 assemblies are dynamic, and driven by reduction in the protein’s NCD, as previously demonstrated using other methods (11, 23). Further, using confocal microscopy and fluorescence recovery after photobleaching (FRAP), we reveal that increasing salt concentration drives reflectin A1 LLPS to form protein-dense liquid condensates whose material properties are tuned by protein NCD. We show that as a function of salt concentration, reflectin assembly is intermediate to LLPS and propose that assemblies and liquid condensates represent stages in a continuum of the same physical process mediated by short-range attraction and long-range repulsion (SALR). We also demonstrate that reflectin A1 phase transitions differ markedly from the recently described process of percolation-coupled phase separation (41). We explore the dynamics of reflectin A1 assemblies and the driving forces of reflectin A1 phase transitions as a biophysical model for Bragg lamellar condensation, which governs their tunable photonic behavior. We also investigate the usefulness of reflectin A1 condensates as a basis for tunable biomaterials by probing their time- and NCD-dependent material properties.

## Results

### Reflectin A1 assemblies coexist with dilute phase

**Figure 1:**
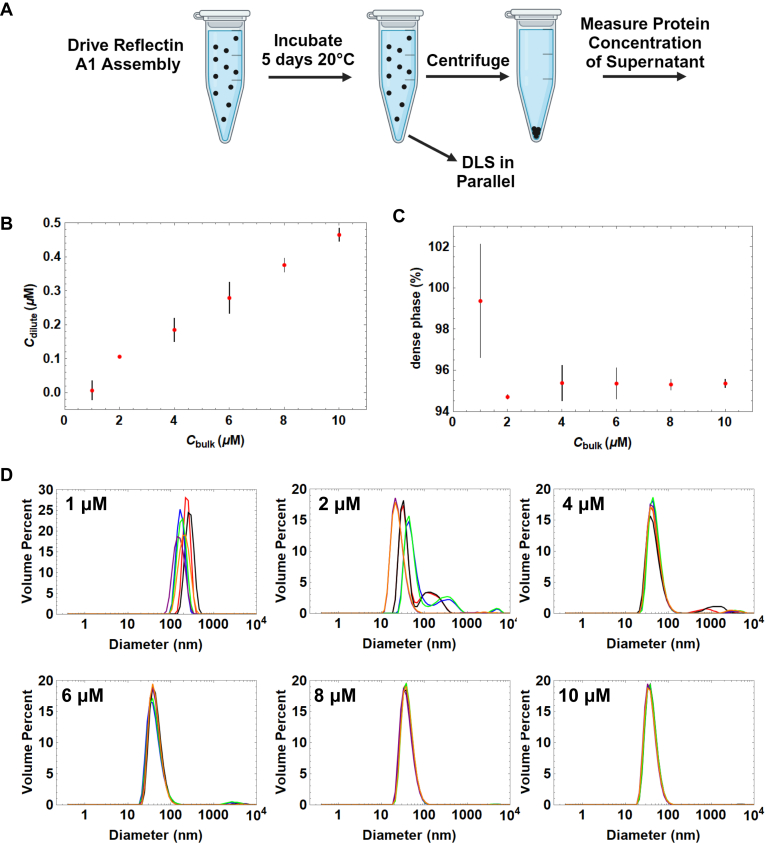
**Bradford assay and DLS of reflectin A1 assemblies as a function of protein concentration.***A*, reflectin A1 was driven to assemble by dilution into MOPS buffer (pH 7 25 mM) and after 5 days dilute phase protein concentration determined by centrifugation assay. DLS of assemblies performed in parallel. *B*, reflectin A1 dilute concentration (*y*-axis) as a function of bulk concentration (*x*-axis). *C*, total protein partitioning into assemblies (*y*-axis) as a function of bulk concentration (*x*-axis). *D*, diameter of reflectin A1 assembly sizes as a function of bulk protein concentration. Data points for (*B* and *C*) represent three experimental replicates with three measurement replicates each. Error bars represent ± 1 SD. For (*D*), three technical replicates of three experimental replicates each are shown as individual lines. *y*-axis is volume percent and *x*-axis diameter in nm. DLS, dynamic light scattering.

Following assembly of reflectin A1, a centrifugation assay was used to demonstrate and analyze the coexistence of a dense phase of assemblies and a dilute phase of monomers or small oligomers (41). Reflectin A1 at an initial concentration of 100 μM in acetic acid buffer (pH 4, 25 mM) was assembled by dilution to into 3-(*N*-morpholino)propanesulfonic acid (MOPS) buffer (pH 7, 25 mM), and after 5 days at 20 °C, samples were centrifuged at 20,000*g* for 6 h to remove assemblies (Fig. 1*A*). The assemblies were incubated for 5 days to ensure that the two phases had reached equilibrium (42). Bradford assays revealed that the protein concentration in the dilute phase, C_*dilute*_, increased in linear proportion to the bulk protein concentration, C_*bulk*_, for C_*bulk*_ between 1 and 10 μM (Fig. 1*B*). Although the protein concentration of the dense assembly phase, C_*dense*_, is unknown, the ratio of moles protein in monomer or small oligomers to moles protein in assemblies was constant for C_*bulk*_ ≥2 μM. This value decreased from 99.4 ± 2.7% for C_*bulk*_ = 1 μM to 94.7 ± 0.1% for C_*bulk*_ = 2 μM and remained relatively constant up to C_*bulk*_ = 10 μM (95.4 ± 0.18%) (Fig. 1*C*). C_dilute_ for reflectin A1 assemblies depends on C_bulk_, which differs from classical LLPS in which C_*dilute*_ increases with C_*bulk*_ until it exceeds the saturation concentration C_*sat*_, after which any additional increase in C_*bulk*_ is partitioned into the dense phase, after which C_*sat*_ = C_*dilute*_ (19, 43).

Reflectin A1 assembly sizes decrease as C_*bulk*_ is increased from 1 to 4 μM (Fig. 1*D*). Notably, the size distribution of assemblies narrows as C_*bulk*_ is increased from 1 to 10 μM (Fig. 1*D*). Assembly sizes range from 70 to 500 nm diam. for C_*bulk*_ of 1 μM. At C_*bulk*_ = 2 μM, size distributions are bimodal with a larger proportion by volume of assemblies in the smaller size distribution (Fig. 1*D*). As C_*bulk*_ is further increased to 10 μM, this bimodality decreases (Fig. 1*D*). Reflectin A1 assembly size as a function of concentration mirrors the dependence of C_*dilute*_ on C_*bulk*_. Partitioning of reflectin A1 into assemblies for C_*bulk*_ ≤2 μM varies from C_*bulk*_ ≥4 μM which is relatively constant (Fig. 1*C*). Assembly sizes for C_*bulk*_ ≤2 μM vary from assembly sizes for C_*bulk*_ ≥4 μM which is then relatively constant (Fig. 1*D*). Importantly, reflectin A1 assembly sizes do not increase with increasing protein concentration over the range analyzed in these conditions.

### FRET reveals reflectin A1 assemblies dynamically exchange protein molecules with dilute phase

**Figure 2:**
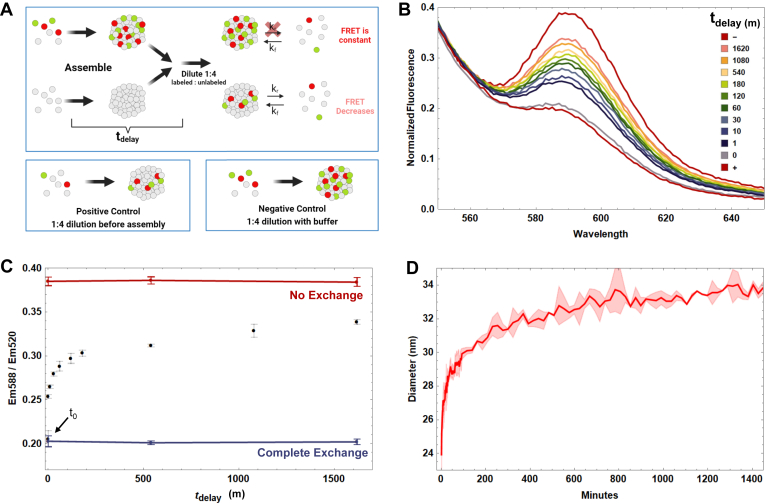
**FRET assay monitors dynamic exchange of reflectin A1 assemblies as a function of time.***A*, design of FRET dilution experiment. Mixtures of 100 μM reflectin A1 containing 5% fluorescein-labeled single cysteine mutant C232S (A1 C199-F) and 5% rhodamine sulfate–labeled C232S (A1 C199-R) were driven to assemble by dilution into MOPS buffer (pH 7, 25 mM). Simultaneously, unlabeled reflectin was driven to assembly under the same conditions. As a function of time postassembly (t_delay_), labeled assemblies were diluted 1:4 with unlabeled assemblies, and then incubated for 24 h. Samples were excited with 488 nm wavelength laser corresponding to the absorption maximum of the donor fluorophore fluorescein and emission spectra from 510 to 700 nm recorded. For the negative control, fluorescently labeled assemblies were diluted 1:4 with buffer only. Reflectin A1 solutions containing 5% A1 C199-R and 5% A1 C199-F were diluted 1:4 with unlabeled reflectin prior to assembly for the positive control. *B*, emission spectra normalized to 520 nm (Em_max_ of fluorescein) as a function of t_delay_, the time between assembly and mixing of labeled and unlabeled assemblies. *C*, FRET emission at 588 nm normalized to 520 nm and plotted as a function of t_delay_. The negative control represents zero dynamic exchange (*red line*) and the positive control represents complete dynamic exchange (*blue line*). *D*, in parallel, assemblies of reflectin A1 were formed by dilution of same protein stock into MOPS buffer (pH 7, 25 mM) and sizes determined by dynamic light scattering. For DLS, data points represent three experimental replicates. Error bars (*C*) and error bands (*D*) represent ± 1 SD.

We initially tested the hypothesis of dynamic exchange by mixing donor-fluorophore–labeled reflectin assemblies with acceptor-labeled assemblies (Fig. S3), but realized this would not distinguish between 1) the bidirectional exchange of protein between reflectin assemblies and 2) unidirectional exchange as nondynamic assemblies form and grow. Therefore, we utilized the following fluorophore dilution scheme: reflectin A1 assemblies labeled with both fluorescein (FRET donor) and rhodamine (FRET acceptor) were diluted with unlabeled A1 assemblies (Fig. 2*A*). A decrease in FRET would represent a decrease in the concentration of fluorophores within the protein assemblies, which would necessitate influx of unlabeled and efflux of fluorescently labeled reflectin A1. After diluting fluorescently labeled reflectin A1 with unlabeled A1 immediately after assembly formation, a decrease in FRET was observed in emission spectra (Fig. 2*B*). Replotting FRET as a function of time demonstrates that the dynamic exchange of reflectin between assemblies slows as the assemblies’ age (Fig. 2*C*). Reflectin A1 assemblies diluted immediately after formation are completely dynamic as FRET decreases to the positive control value (Fig. 2*C*), while only 19% still dynamically exchanging for assemblies aged 24 h before dilution. Reflectin A1 assembly sizes increased over the same time scale as FRET measurements of dynamic exchange (Fig. 2*D*). Initial assembly diameter was 24 ± 1.6 nm, and the assemblies grew to 33.9 ± 0.35 nm diam. after 24 h.

Additionally, analysis of changes in FRET as a function of time for freshly formed assemblies was used to analyze the rate of dynamic exchange between assemblies (Fig. S4). The FRET signal decreases from approximately that of the negative control (no exchange) and approached the positive control (complete exchange), while the rate of dynamic exchange decreased with time. However, because reflectin A1 assemblies continued to grow for up to 1400 min (Fig. 2*D*), we suspected that the time scale associated with dynamic exchange would be of similar magnitude and that the kinetic FRET experiment was not long enough to capture complete exchange.

### Increasing salt concentration drives LLPS of reflectin A1

**Figure 3:**
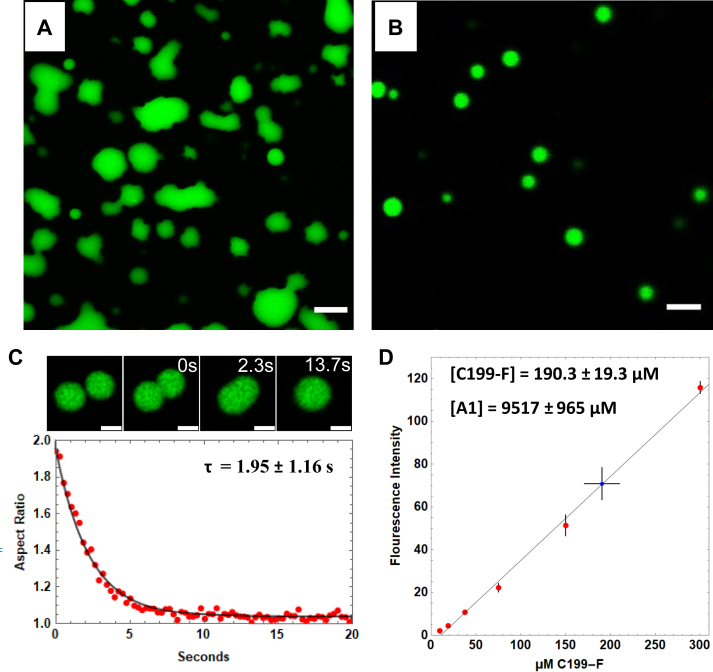
**Confocal microscopy of fluorescently labeled reflectin A1 droplets.** One hundred micromolars reflectin A1 containing 5% fluorescently labeled A1 C199-F was diluted to a final protein concentration of 4 μM in acetic acid buffer (pH 4, 25 mM) in 250 mM NaCl. *A*, reflectin liquid condensates wet untreated glass coverslips. *B*, condensates form spherical droplets on PEG-passivated coverslips. *C*, condensates fuse and relax to sphericity. The change in aspect as a function of time fits to an equation of exponential decay yielding the relaxation time τ. *D*, reflectin A1 interdroplet, or dense phase, protein concentration is 9517 ± 965 μM, or 416 ± 42 mg/ml. Monomeric standards (*red*) of 100% A1 C199-F in acetic acid buffer (pH 4, 25 mM) were used to relate fluorescence intensity to concentration. Intensities of droplets of 2% C199-F were averaged (*blue*), (n = 147 droplets from five experiments). Error bars = ± 1 SD. Scale bars for (*A* and *B*) represent 5 μm and (*C*) = 2 μm.

The dilution of 100 μM reflectin A1 containing 5% single cysteine mutant A1 C232S covalently labeled with fluorescein (referred to as A1 C199-F to denote the amino acid location of fluorescein) into acetic acid buffer (pH 4, 25 mM) in 250 mM NaCl forms liquid condensates that wet untreated glass coverslips (Fig. 3*A*). Wetting is prevented using PEG passivated coverslips (Fig. 3*B*), causing the condensates to remain spherical and undergo fusion with relaxation to sphericity upon contact (Fig. 3*C*). The rate of change of aspect ratio of newly fused droplets fits well with an equation of exponential decay that is commonly used to characterize liquid droplet dynamics (37, 44) (Fig. 3*C*), yielding a relaxation time *τ* of 1.95 ± 1.16 s. Surface wetting and relaxation of newly fused droplets indicate that the condensates exhibit surface tension which is characteristic of a liquid (19, 45).

The results described above show that under these conditions, reflectin A1 undergoes LLPS to form protein-dense and protein-dilute liquid phases. Comparison of the mean fluorescence intensity of reflectin A1 containing 2% A1 C199-F in acetic acid buffer (pH 4 25 mM) in 250 mM NaCl to that of 100% A1 C199-F monomeric standards in acetic acid buffer (pH 4 25 mM) determined the dense phase protein concentration to be 9517 ± 965 μM, or 416 ± 42 mg/ml (Fig. 3*D*). This concentration is within the range of dense phase protein concentrations reported for many other liquid protein condensates (46, 47, 48) and is similar to reflectin protein concentration within the Bragg lamellae of *Doryteuthis opalescens* iridocytes, which was previously estimated to be 381 mg/ml (12). Thus, compared to the assemblies previously observed in low salt conditions, reflectin condensates may represent a more physiologically relevant biophysical model for investigating reflectin protein interactions.

### Reflectin A1 phase diagram shows reciprocal relationship between NaCl concentration and protein NCD

**Figure 4:**
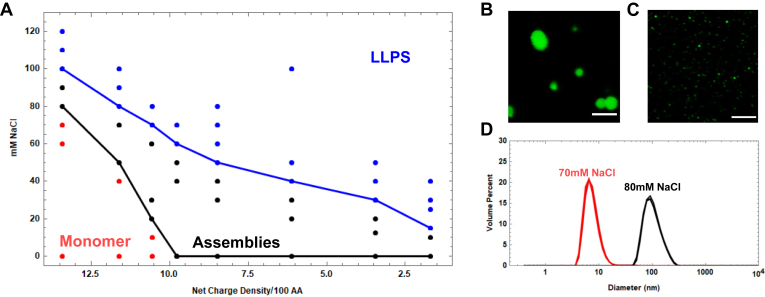
**Phase diagram of reflectin A1 as a function of NaCl concentration and calculated protein net charge density.***A*, reflectin A1 can exist as monomer (*red*) or assemblies (*black*), or liquid-like condensates (*blue*). *B*, liquid-like droplets were distinguished from (*C*) light-resolvable assemblies by size discrepancy as well as surface wetting and droplet fusion. *D*, assemblies were distinguished from monomers by using DLS to measure particle size distributions. Three DLS replicates at each condition are shown cumulatively. At pH 4 in 70 mM NaCl, reflectin A1 exists as 7.9 ± 3.5 nm diam. monomers, while at pH 4 in 80 mM NaCl the protein forms 106 ± 39 nm diam. assemblies. Scale bars represent 5 μm. At high pH, deprotonation of histidines decreases the protein net charge density per 100 amino acids. Net charge density is calculated from the pKa of each amino acid residue of reflectin A1 at each experimentally manipulated pH (11). DLS, dynamic light scattering.

To investigate the role of protein NCD and salt concentration on reflectin A1 phase transitions, as well as the potential for the use of reflectin A1 in tunable biomaterials, we determined the phase diagram for reflectin A1 as a function of ionic strength and NCD, which is defined by three species: monomer, assembly, and liquid condensate (Fig. 4*A*). DLS was used to determine the boundary between monomer and assembly using the previously established R_h_ of reflectin A1 monomer (17, 21, 23) (Fig. 4*D*). The boundary between reflectin A1 assemblies and liquid condensates was determined by confocal microscopy of reflectin A1 containing 5% A1 C199-F. Droplets with the previously mentioned liquid characteristics (Fig. 4*B*) were clearly distinguished from observable and subresolution assemblies (Fig. 4*C*). As the liquid phase boundary was approached by increasing ionic strength at a given pH (and its corresponding protein NCD), assemblies increased from subresolution to microscopically resolvable sizes (Fig. S6). Upon crossing the liquid phase boundary, protein partitioning into the dense phase increased markedly (Fig. S6). Additionally, the presence of slow modes in the DLS autocorrelation functions confirmed the observed microscopic distinctions between size-stable assemblies and liquid condensates at high NCD. Droplets with the previously mentioned liquid characteristics were detected microscopically upon dilution of reflectin A1 into 100 mM NaCl in acetic acid buffer (pH 4, 25 mM) (Fig. S7*A*), with DLS autocorrelation slow modes present and increasing with time (Fig. S7*B*), which is characteristic of LLPS (41). In marked contrast, the same analyses in 90 mM NaCl in acetic acid buffer (pH 4 25 mM) failed to produce resolvable droplets (Fig. S7*A*), and the DLS autocorrelation functions did not change with time nor display slow modes (Fig. S7*B*).

The decreasing slopes of both phase boundaries at high NCDs reveal that as the cationic charge of the protein is progressively neutralized (NCD is decreased), a progressively lower ionic strength is required to drive assembly or LLPS, consistent with the suggestion that pH titration and charge screening from salt act similarly (21). For lower protein NCDs (pH 5.5–8), reflectin A1 is not monomeric at any salt concentration in 25 mM buffers. For all protein NCDs tested, the assembly/LLPS boundary shares the same trend of decreasing protein NCD requiring decreasing ionic strength to drive LLPS (Fig. 4*A*). This trend at moderate salt concentrations, where Debye lengths are similar to the distances of ionic interactions (43, 49), further supports the suggestion that ionic charge screening from salt drives both assembly and LLPS (50, 51, 52). In addition to increasing the ionic strength’s contribution to screening of repulsive charges (23), surface tension will also increase, thereby reducing protein solubility by increasing the contribution of any hydrophobic drivers of assembly and LLPS.

### Salt drives LLPS by increasing both the hydrophobic effect and anionic screening

**Figure 5:**
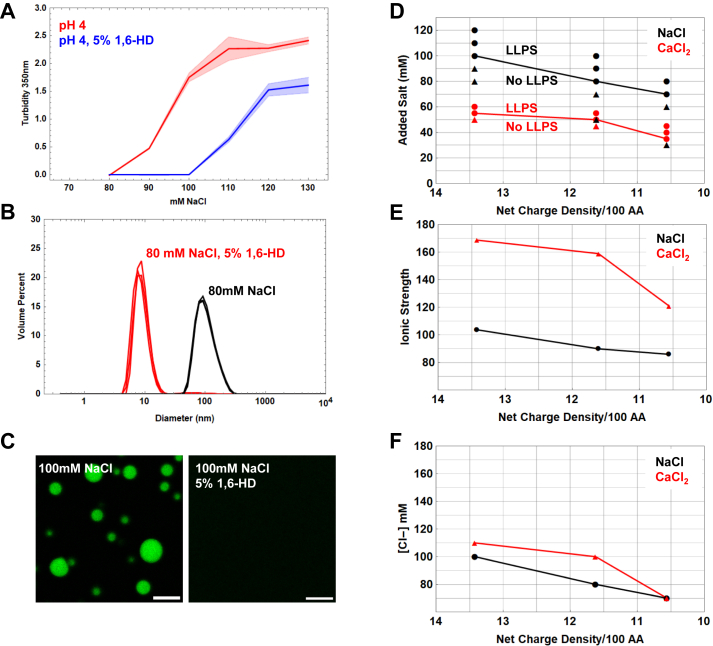
**Effects of 1,6-hexanediol and anion concentration on reflectin A1 phase transitions.***A*, reflectin A1 was diluted into 80 to 130 mM NaCl (*red*) with 5% 1,6-HD (*blue*) and turbidity measured at 350 nm. *B*, DLS of dilution of reflectin A1 into pH 4 80 mM NaCl (*black*) results in 106 ± 39 nm diam. assemblies and dilution into pH 4 80 mM NaCl, 5% 1,6-HD with yields monomers of 9.0 ± 2.6 nm diam. *C*, dilution of reflectin A1 into pH 4 100 mM NaCl forms droplets but the inclusion of 5% 1,6-hexanediol prevents droplet formation. Turbidity measurements are shown as the average of three experimental replicates with three measurement replicates each; error bands represent ± 1 SD. The same numbers of DLS replicates are shown cumulatively, and images in (*C*) are characteristic of three experimental replicates. The scale bar represents 10 μm. *D*, liquid phase boundaries determined presence or absence of droplets upon addition of NaCl (*black*) or CaCl_2_ (*red*). *E*, same data as (*D*) displayed as a function of ionic strength and (*F*) Cl^-^ concentration. *x*-axis for (*D*, *E*, and *F*) is calculated protein net charge density using the method described in Figure 4. Data for NaCl addition is the same as that used for Figure 4. For all experiments 2 μl of 100 μM reflectin A1 in acetic acid buffer (pH 4 25 mM) was diluted into 48 μl of respective buffer. DLS, dynamic light scattering.

To determine the possible role of the hydrophobic effect in driving reflectin A1 assembly and LLPS, the protein’s behaviors were compared in the presence and absence of 5% 1,6-hexanediol (1,6-HD). This water-soluble aliphatic alcohol lowers the surface tension of water (35) and has been shown to dissolve liquid protein condensates that are formed by the hydrophobic effect (35, 53, 54, 55). Turbidity measurements of reflectin A1 diluted into acetic acid buffer (pH 4, 25 mM) from 80 to 100 mM NaCl demonstrate that 5% 1,6-HD inhibited the salt-driven formation of large, light-scattering particles of reflectin A1 (Fig. 5*A*). In the absence of 1,6-HD, turbidity increased at 90 mM NaCl and was approximately 2.5 at 130 mM NaCl. In contrast, in the presence of 5% 1,6-HD, turbidity increased at 110 mM NaCl and reached only 1.5 (Fig. 5*A*). Similarly, while the dilution of reflectin A1 into 80 mM NaCl at pH 4 formed 52.7 ± 18.3 nm diameter assemblies, the inclusion of 5% 1,6-HD prevented this assembly (Fig. 5*B*). In 100 mM NaCl at pH 4, reflectin A1 formed liquid droplets, but the inclusion of 5% 1,6-HD abrogated LLPS and droplets were not observed (Fig. 5*C*). Further, DLS analyses reveal slow modes in the autocorrelation function at 100 mM NaCl at pH 4 which are no longer seen after the inclusion of 5% 1,6-HD (Fig. S7*C*). These results indicate that increasing ionic strength drives reflectin A1 assembly and LLPS in part by increasing the contribution of the hydrophobic effect. This would be expected because of the large gain in entropy typically resulting from burying hydrophobic side chains away from water (56, 57, 58) and LLPS is primarily a segregative process (19) driven by differences in polymer and solvent polarity.

It has been previously demonstrated that the sizes of reflectin A1 assemblies formed by addition of salt are well predicted by the concentration of anions (23). To investigate if this relationship is also true for salt-induced LLPS of reflectin A1, the liquid phase boundary of reflectin A1 was determined as a function of CaCl_2_ concentration in addition to NaCl concentration for pH values 4 to 5 (Fig. 5*D*). The liquid phase boundaries as a function of anionic concentration (Fig. 5*F*) differ by 9.6% for pH 4, 21% for pH 4.5, and are identical for pH 5. In comparison, the liquid phase boundaries as a function of ionic strength (Fig. 5*E*) vary by 62% for pH 4, 48.9% for pH 4.5, and 20.4% for pH 5. Therefore, the concentration of anionic species better describes the liquid phase boundary determined by addition of NaCl and CaCl_2_ than does ionic strength, which strongly suggests that electrostatic screening of cationic Coulombic repulsion within and between reflectin A1 molecules contributes to the drive of reflectin A1 to undergo LLPS. Screening of long-range repulsion at high protein NCDs would allow the short-range attractive forces to drive reflectin A1 folding, assembly, and LLPS. We conclude that increasing salt concentration and reducing protein NCD drive reflectin A1 phase transitions by the same physical processes: both reduce protein solubility as well as neutralize protein NCD.

### Reflectin A1 condensates demonstrate liquid properties up to 96 h

**Figure 6:**
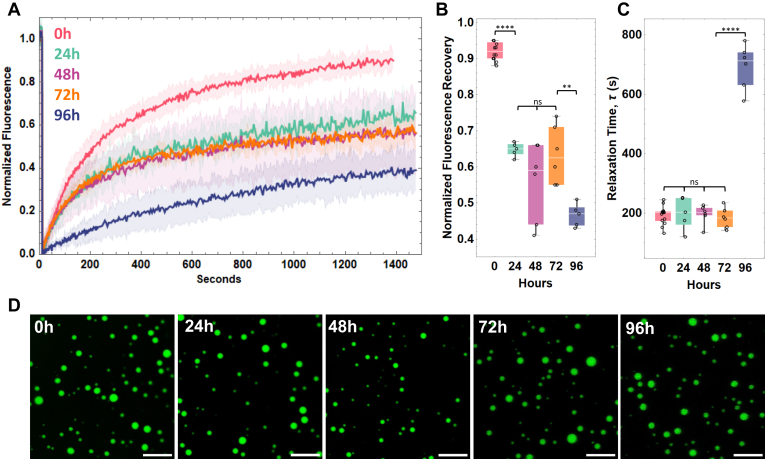
**Time-dependent diffusivity of reflectin A1 condensates.***A*, FRAP of reflectin A1 liquid-like condensates in pH 4 25 mM acetic acid 250 mM NaCl for 0 to 4 (*red*), 24 (*green*), 48 (*purple*), 72 (*orange*), and 96 h (*blue*). *B*, data were corrected for photobleaching and fully normalized fluorescence intensity of bleach spots plotted as a function of time. *C*, fitting of individual experiments to an exponential decay equation yields the characteristic relaxation time τ. Statistical significance determined by one-way ANOVA testing. Box and whisker plots display the median (*center line*), second and third quartile (*solid box*), and the complete range (*black fences*) of the data. *D*, droplet morphologies for 0 to 96 h. N = 12 for 0 h, N = 5 for 24 h, N = 6 for 48 h, N = 6 for 72 h, and N = 6 for 96 h. FRAP, fluorescence recovery after photobleaching.

FRAP was used to investigate the aging of reflectin A1 droplet dynamics (Fig. 6*A*) over time scales that far exceed *in vivo* reflectin activation (12), but are relevant for the use of reflectin A1 as a tunable biomaterial. The percent fluorescence recovery from FRAP of reflectin A1 condensates, which represents the proportion of the liquid-like population of the dense phase, in pH 4 25 mM acetic acid 250 mM NaCl decreased significantly from 0 to 24 h, does not change significantly from 24 to 72 h, and then decreased over from 72 to 96 h. (Fig. 6*B*). Remarkably, *τ* did not change significantly from fresh droplets to 72 h, but then increased drastically from 72 to 96 h (Fig. 6*C*). Reflectin A1 droplets showed no visible changes in morphology such as jaggedness or fibrilization for the duration of the experiment (Fig. 6*D*). Despite the stability of τ in these conditions, droplet size did not change significantly over 96 h suggesting that Ostwald ripening was arrested (Fig. 6*D*) (59, 60, 61). The time-dependent decrease in both the liquid-like proportion of proteins and the diffusivity of this population within reflectin A1 droplets may be due to an increase in noncovalent interactions and/or progressive dehydration (62, 63).

### NCD and ionic strength tune reflectin liquid condensate dynamics

**Figure 7:**
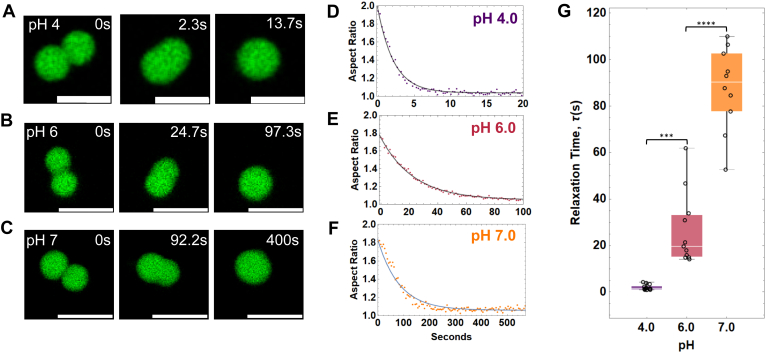
**Quantification of reflectin A1 condensate fusion dynamics.** Aspect ratios of newly fused droplets in 250 mM NaCl at pH 4, 6, and 7 were calculated from videos. Representative time series of droplet fusion and relaxation at (*A*) pH 4, (*B*) pH 6, and (*C*) pH 7. Aspect ratio as a function of time was fit to an exponential equation of decay to determine τ for (*D*) pH 4, (*E*) pH 6, and (*F*) pH 7. *G*, changes in aspect ratio over time between each pH were found to be statistically significantly different using a one-way ANOVA test. Box and whisker plots display the median (*center line*), second and third quartile (*solid box*) and the complete range (*black fences*) of the data. N = 23 for pH 4, n = 12 for pH 6, and n = 10 for pH 7. Scale bars represent 5 μm. (*A*) is the same representative image used in Figure 3*C*.

Motivated by the potential use of reflectin A1 for tunable biomaterials, we used FRAP and droplet fusion dynamics to determine that the liquidity of reflectin A1 condensates is tuned by protein NCD. Videos of reflectin A1 condensates in 250 mM NaCl at pH 4, 6, and 7 (Fig. 7, *A*–*C*) were used to determine the change in aspect ratio of newly fused droplets as a function of time. Fitting these data to an equation of exponential decay (Fig. 7, *D*–*F*) reveals that relaxation time *τ* increased from 1.95 ± 1.16 s at pH 4 to 36.95 ± 29.32 s at pH 6, and ultimately to 84.86 ± 22.77 s at pH 7 (Fig. 7*G*). Droplet fusion and relaxation rates increased by almost two orders of magnitude from pH 4 to pH 7, demonstrating that liquidity (44) of reflectin A1 condensates decreases as protein NCD decreases.

**Figure 8:**
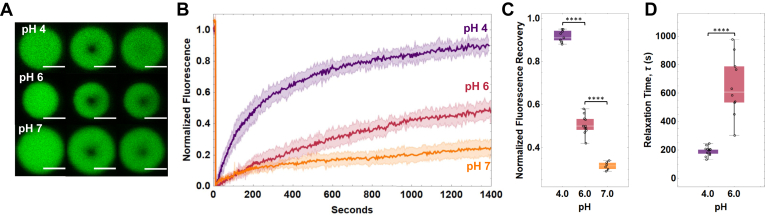
**FRAP of reflectin A1 condensates at pH 4, 6, and 7 in 250 mM NaCl.***A*, characteristic images of droplets before, immediately, and 1400 s post photobleaching for pH 4, 6, and 7. *B*, data were corrected for photobleaching and fully normalized fluorescence intensity of bleach spots plotted as a function of time. *C*, percent fluorescence recovery for pH 4, 6, and 7. *D*, fitting of individual experiments to an exponential decay equation yields the characteristic relaxation time τ. τ for pH 4 and pH 6, as well as percent recovery for all pHs were found to be statistically significant using a one-way ANOVA test. Box and whisker plots display the median (*center line*), second and third quartile (*solid box*), and the complete range (*black fences*) of the data. Accurate τ values for pH 7 could not be obtained by fitting. N = 12 for pH 4, n = 11 for pH 6, and n = 7 for pH 7. Error bands in FRAP plots represent ± 1 SD. Scale bars represent 5 μm. FRAP, fluorescence recovery after photobleaching.

FRAP analyses of reflectin A1 droplets in 250 mM NaCl at pH 4, 6, and 7 (Fig. 8, *A* and *B*) demonstrate a dependence of percent fluorescence recovery and *τ* on protein NCD. The proportion of mobile component of the droplets decreases as pH increases (protein NCD decreased) (Fig. 8*C*), indicating that the population of liquid-like protein molecules in the droplet is decreasing. Fluorescence recovery rates *τ* increase from 192.3 ± 31.1 s at pH 4 to 641.6 ± 220.2 s at pH 6, and at pH 7 the dynamics within reflectin condensates were too slow to determine accurate *τ* values (Fig. 8*D*). This dependence on protein NCD could be due to more rapid aging of noncovalent protein interactions at lower protein NCDs (higher pHs) than at higher protein NCDs (lower pHs), as more extensive protein–protein interactions would be expected for decreasing Coulombic repulsion at low protein NCDs. It also is possible that reflectin A1 condensates formed at low protein NCDs are less hydrated and more protein dense than condensates formed at high protein NCDs, further contributing to the slowing of condensate dynamics. Reflectin A1 droplets showed slower and incomplete fluorescence recovery than many other *in vitro* and *in vivo* protein systems (33, 37, 38, 64, 65, 66, 67, 68, 69).

## Discussion

Reflectin A1’s NCD has been shown to finely control the protein’s assembly sizes, and it has been hypothesized that progressive neutralization of the protein’s excess cationic charge, reducing Coulombic repulsion both within and between reflectin molecules, mediates this relationship (11), both physiologically in response to neuronally activated phosphorylation (6, 10) and *in vitro* by various surrogates (11, 21, 22). Here, we extend these results to demonstrate that further reduction of Coulombic repulsion by ionic screening drives reflectin A1 to undergo LLPS into liquid condensates. Notably, as salt concentration increases, assemblies increase in size (Fig. S6) (23) and reflectin A1 assembly always precedes LLPS, suggesting that reflectin A1 assembly and LLPS are stages of the same physical path (45, 59). The dependence of the liquid phase boundary of reflectin A1 on the concentration of anions at high protein NCDs (Fig. 5) supports the hypothesis that ionic screening of Coulombically repulsed cationic reflectin A1 allows for attractive forces (*e.g.*, cation-pi, sulfur-pi, hydrophobic effect) to drive LLPS of reflectin A1. Importantly, this matches and extends the recent demonstration that salt induces reflectin A1 assembly by ionic screening (23), and both mechanisms are consistent with the findings that the extent of reduction of reflectin A1’s NCD by phosphorylation, pH titration, genetic engineering, or electroreduction proportionally control reflectin A1 assembly sizes (11). The phase behavior of reflectin A1 can be compared with that of other proteins exhibiting both assembly and LLPS behavior (41, 70, 71), notably FUS cluster formation in a prepercolation state of percolation-coupled phase separation. That behavior is not consistent with our observations of reflectin A1 assemblies, in which 1) 95 to 99% of bulk reflectin A1 protein is found in assemblies; 2) reflectin A1 assembly sizes are relatively monodisperse and display a normal distribution as detected by DLS in our observations and previously (11, 17, 23); and 3) reflectin A1 assembly sizes did not increase with increasing concentration for the range of 1 to 10 μM. Contrary to the behavior of reflectin A1, solutions of proteins FUS, TDP-43, BrD4, Sox2, and A11 undergo LLPS under low salt concentrations (<50 mM KCl), exist as a single phase at intermediate salt concentrations (125 mM–1.5 M KCl), and are prevented from undergoing LLPS by the disruption of attractive ionic protein interactions by electrostatic screening at intermediate salt concentrations (53). Hence, it is not without precedent that intermediate salt concentrations [concentrations where the Debye distance is similar to that of electrostatic interactions between amino acid side chains (43, 49)] can dictate liquid phase transitions of proteins by screening ionic interactions, albeit with reflectin it is the screening of repulsive electrostatic interactions that drives assembly and LLPS.

Both reflectin A1 assembly and LLPS are driven by increasing the relative contribution of the hydrophobic effect. Previously, shifts in fluorescence spectra of the native tryptophan residues in reflectin A1 as well as hydrophobic binding of the fluorophore 8-anilinonapthalene-1-sulfonic acid demonstrated a net transfer of these fluorophores into a more hydrophobic environment upon reflectin A1 assembly (17). Although the primary sequence of reflectin A1 does not have any significant stretches of hydrophobic amino acids, computational analyses of hydrophobic moments, which are the net amphiphilicity of a given protein segment as a function of the angle between successive side chains (72), show the highly conserved RMs have maximal hydrophobic moments at side chain angles consistent with *α*-helical (100°) and *β*-sheet (160°) secondary structures (16), and these secondary structures emerge upon folding and assembly of reflectin A1 (11, 22). These results suggest that the emergent hydrophobicity upon secondary structure formation in the RMs may provide a major hydrophobic contribution to folding, assembly and LLPS of reflectin A1.

Kinetic analysis of FRET reveals that the rate of dynamic exchange decreases over time (Fig. S4). This is likely due in part to the decrease in surface area available for exchange as assemblies grow larger (60), but also due to a decrease in the extent of the population that participates in dynamic exchange over time as demonstrated by the FRET time series analyzed as a function of assembly age. This time series and corresponding size distribution analysis show that the decrease in the extent of dynamic exchange mirrors the rate of assembly growth. Assuming that the concentration of FRET pairs in the dilute phase is lower than that in the dense assembly phase, the formation of a nondynamic population or outer region of assemblies from one-way transport of particles from the dilute phase into the dense phase would not contribute either to a dilution of FRET pairs in the assemblies nor to a decrease in the FRET signal over the time observed (Fig. 2*C*). This conclusion is further supported by the negative controls for both the FRET time series (Fig. 2, *A* and *C*) and kinetic analysis of FRET (Fig. S4); both demonstrate no significant decrease in the FRET signal over time. It is unknown if the interior of assemblies is freely accessible to solvent and smaller protein particles in the dilute phase, or if smaller protein particles must diffuse through a molten outer region of assemblies to access the center. Because the FRET signal does not provide information regarding the structure responsible for the decrease in extent of dynamic exchange, we can only conclude that the time-dependent decrease in the rate of dynamic exchange correlates with the decreasing rate of assembly growth. This is consistent with an arrest of Ostwald ripening, a mechanism by which condensates increase in size by exchange of particles through the dilute phase (45).

Thermodynamic equilibrium, as opposed to kinetic arrest or glassification, could define reflectin A1 assembly size by a trade-off between short range attractive forces and long-range repulsive forces between monomers (SALR) (73). The electrostatic repulsion between cationic reflectin A1 proteins can be described as a long-range repulsion that, in continual balance with short-range attractions (including those of hydrophobic, cation–pi, and sulfur–pi interactions), define both the protein’s assembly sizes and phase transitions. Thus, LLPS of reflectin A1 may represent such a state in which the electrostatic repulsive energy barrier is no longer sufficient to restrict assembly size. Excitingly, computational modeling of reflectin A1 assemblies as a function of protein NCD (*via* pH titration) well describes A1 assembly size distributions as SALR-dictated equilibrium clusters (40). A colloidal model was developed in which reflectin monomers interact *via* a short-range attractive and long-range repulsive pair potential (SALR), with parameters of the model calibrated with data from the experimental short-angle X-ray scattering analyses of reflectin A1 assemblies in which size was tuned by pH titration. This model predicted that reflectin A1 assemblies are dynamic equilibrium clusters, successfully predicting assembly sizes as a function of protein NCD that were consistent with previous DLS observations (11, 40). Decreasing the modeled long-range repulsion increased equilibrium cluster size until LLPS occurred. Further, this analysis predicts a phase diagram for reflectin A1 as a function of protein NCD that agrees with that presented here for the protein concentrations used in our experiments. However, because reflectin A1 phase behavior is likely influenced by the valency of interprotein interactions, a possible shortcoming of relating this behavior to that of simple colloidal systems is that these do not depend on such valency—unlike many other proteins that undergo physiologically relevant LLPS (19, 45, 59, 73, 74, 75, 76, 77, 78, 79).

The complex phase behavior of reflectin A1 adds to our understanding of the possible states of matter in the Bragg lamellae in the tunable iridocytes of *D. opalescens. In vivo*, iridocyte activation and change in reflectance in response to acetylcholine occurs on the timescale of 10 s (5, 10), and our FRET observations show little decrease in the rate or extent of dynamic exchange between assemblies over the same time (Fig. 2). This demonstrates that reflectin A1 assemblies can remain dynamic over the physiologically relevant time. The protein concentration in reflectin condensates was found to be 416 ± 42 mg/ml (Fig. 3*D*), similar to the previous estimate of the *in vivo* lamellar protein concentration of 381 mg/ml (12). Our finding that progressive neutralization of reflectin protein NCD drives a phase transition from multimeric assemblies to a dense liquid phase parallels observations of the reflectin-containing iridocytes and leucophores in the living tissue and observations of reflectin’s behavior in the isolated Bragg lamellae. Upon acetylcholine activation, reflectin in the iridocyte Bragg lamellae and leucophore vesicles undergoes a phase transition from 10 to 50 nm diameter particles into a dense liquid (5, 13, 39). Bragg lamellae isolated from activated iridocytes of the Loliginid squid *Lollinguncula brevis* showed liquid properties such as surface wetting and deformability, in contrast to those isolated from nonactivated iridocytes (39).

Expression of reflectin A1 from *Doryteuthis pealeii* (80) and individual expression of reflectins A1, A2, B, and C from *D. opalescens*, both in HEK 293T cells, yielded intracellular spherical puncta consistent with LLPS (81). In tunable iridocytes, a liquid phase transition would maximize the lamellar protein density relative to a solution of assemblies and therefore enhance the refractive index contrast resulting from activation, while maintaining a dynamic environment essential for the controlling activities of reflectin-specific kinases and phosphatases. Decreasing the protein’s NCD by phosphorylation could provide a mechanism for tuning lamellar dehydration and the consequent photonic behavior (7, 8, 12) by controlling the dense phase protein concentration. Further, Bragg lamellae in tunable iridocytes from *D. opalescens* are enriched in reflectins B and C and relatively low in reflectin A2 compared to nontunable iridocytes from the same species (6). It remains to be seen if reflectins A2, B and C also undergo LLPS in protein NCD-dependent manner, and if these species affect the liquid phase boundary and condensate properties of reflectin A1. Nonetheless, the finding that reflectin A1 can be driven to LLPS by changing protein NCD and ionic strength, and that the liquid properties of these condensates is controlled similarly, illuminates a significant extension of both our understanding of Bragg lamellar condensation and the remarkable tunability of the reflectin system.

## Experimental procedures

### Protein expression and purification

Reflectins A1, A1-C232S, and A1-6E were purified as previously described (11). Codon-optimized sequences of reflectins A1 and A1-C232S were cloned into pj411 plasmids. Proteins were expressed in Rosetta 2 (DE3) cells grown in 1 or 2 l LB cultures at 37 °C from plated and sequenced transformants in the presence of 50 mg/ml kanamycin. Expression was induced at an absorbance of 0.6 to 0.7 with 5 mM IPTG. After 16 h, cultures were pelleted by centrifugation and frozen at −80 °C. Reflectins were expressed in inclusion bodies, which were purified using BugBuster medium (Novagen, Inc) per manufacturer protocol, then resolubilized in 8 M urea 5% acetic acid. Reflectins were purified using cation exchange with a 10 ml Hitrap cation-exchange column (Cytiva) and eluted using a step gradient (0% to 7% to 10%) of 5% acetic acid, 6 M guanidinium chloride. Reflectins eluted at 10% eluting buffer. Purity of collected fractions was determined by A260/A280 (0.55–0.57) and SDS-PAGE, which were then pooled and concentrated. Concentrated reflectin was loaded onto reverse-phase HPLC Xbridge 4.6 ml C4 column (Waters) equilibrated with 10% acetonitrile with 0.1% TFA and eluted over a gradient of 100% acetonitrile 0.1% TFA. Fractions were frozen at −80 °C or shell-frozen using an ethanol and dry ice bath, lyophilized, and stored at −80 °C until solubilization. Purity was determined by SDS-PAGE and A260/A280. Lyophilized reflectin was solubilized using 0.22 μm-filtered acetic acid buffer (pH 4 25 mM) and dialyzed using two changes (12 h each) of 1000X sample volume of the same buffer at 4 °C. Protein concentration was calculated using absorbance at 280 nm and molar absorption coefficients for each protein (reflectin A1: 120685 M^−1^ cm^−1^, reflectin A1 C232S: 120560 M^−1^ cm^−1^). Protein stock was centrifuged at 18,000*g* for 15 min at 4 °C prior to use in all assays. Protein stocks were stored at 4 °C between uses.

### Bradford assay

0.5, 1, 2, 3, 4, and 54 μl of 100 μM reflectin A1 in acetic acid buffer (pH 4 25 mM acetic acid) were driven to assembly by dilution into freshly 0.22 μm-filtered MOPS buffer (pH 7.0 25 mM) to a total volume of 50 μl and final protein concentrations of 1, 2, 4, 6, 8, and 10 μM, and then incubated at 20 °C for 5 days. Samples were then centrifuged at 20 °C, 20,000*g* for 6 h, and a small portion of supernatant immediately removed, combined with Bradford dye and absorbances at 595 nm recorded after 30 min. A serial dilution of bovine serum albumin standard was used to create a standard curve. For the Bradford assay, experimental data points represent averages of three experimental replicants with three measurements each. In parallel, centrifugation was omitted, and sizes were determined by DLS using a Malvern Zetasizer Nano. All DLS measurements were replicated with three individual experiments, each with three technical replicates spanning 5 min.

### Fluorescent labeling

Reflectin A1-C232S was covalently labeled using cysteine-specific fluorescein- or rhodamine-methanethiosulfonate (FMTS or MTSR). Fifty micromolars protein was incubated with 500 μM FMTS or MTSR in acetic acid buffer (pH 4, 25 mM) with gentle orbital stirring for 4 h room temperature then overnight at 4 °C. Labeled reflectins were concentrated using 10 K molecular weight cut off Amicon spin filters and excess label removed using the previously described HPLC method (11). Labeling efficiencies for FMTS and MTSR were 100%.

### Förster resonance energy transfer

For FRET dilution experiments, 100 μM Reflectin A1 and 100 μM reflectin A1 containing 5% C199-F and 5% C199-R were separately but simultaneously diluted into MOPS buffer (pH 7, 25 mM) to final protein concentrations of 4 μM. For kinetic analysis, fluorescently labeled reflectin A1 assemblies were then diluted 1:5 with unlabeled assemblies in the cuvette. Immediately, samples were excited at 488 nm and emissions of 520 and 588 nm were recorded. For FRET time series, fluorescently labeled reflectin A1 assemblies were then diluted 1:4 with unlabeled reflectin A1 assemblies after the designated time t_delay_ and incubated in protein LoBind tubes at 20 °C for 24 h before measurements. Using a Cary Eclipse fluorescence spectrometer (Agilent Technologies), samples were excited at 488 nm and emission spectra recorded from 510 to 700 nm. Each data point is the average of three experimental replicates. For the positive control, solutions of 100 μM A1 and 100 μM A1 containing 5% each of C199-R and C199-F in acetic acid buffer (pH 4, 25 mM) were mixed and then assembled by dilution into MOPS buffer (pH 7, 25 mM). For the negative control, fluorescently labeled assemblies were formed as per the experimentals but diluted 1:5 with MOPS buffer (pH 7, 25 mM). All spectra were normalized to 520 nm (Em_max_ of fluorescein).

For mixing of assemblies separately labeled with donor and acceptor fluorophores, 100 μM reflectin A1 containing 5% C199-F and 100 μM reflectin A1 containing 5% C199-R were separately but simultaneously driven to assembly by dilution into MOPS buffer (pH 7, 25 mM) to final protein concentrations of 4 μM. After a designated time, fluorescein-labeled and rhodamine-labeled A1 assemblies were mixed and treated identically to samples in the dilution experiment. For the positive control a solution of 100 μM reflectin containing both 5% C199-F and 5% C199-R was driven to assembly. The negative control mixed assemblies containing 5% C199-R with assemblies containing 5% C199-F 24 h after assembly and measured immediately upon mixing. All buffers were freshly 0.22 μm-filtered before use.

### Phase diagrams

To determine the LLPS boundary, 0.6 μl 100 μM reflectin containing 5% fluorescein labeled protein (A1 C199-F) in acetic acid buffer (pH 4 25 mM acetic acid) were diluted into 9.4 μl of respective buffers to a final protein concentration of 4 μM, tap mixed, and incubated for 10 min using 0.5 ml protein LoBind tubes. The solutions were pipetted onto cleaned glass coverslips and imaged immediately after deposition using Leica SP8 resonant confocal microscope with 63X objective (NRI-MCDB Microscopy Facility). To determine the boundary for transition from monomer to assembly, 2 μl of 100 μM reflectin in acetic acid buffer (pH 4 25 mM acetic acid) were diluted into 48 μl respective buffer to a final protein concentration of 4 μM and incubated as above. Sizes were determined by DLS using a Malvern Zetasizer Nano. All DLS measurements were replicated with three individual experiments with three technical replicates spanning 15 min each. All buffers were freshly 0.22 μm-filtered before use.

### Turbidity assays

Two microliters of 100 μM reflectin in acetic acid buffer (pH 4 25 mM acetic acid) were diluted into 48 μl of respective buffer in the measurement cuvette and tap mixed. After 5 min of incubation at 25 °C, three replicant measurements were recorded. This was repeated for a total of three experimental replicates.

### Glass coverslip passivation

For FRAP and droplet fusion analyses, glass coverslips were passivated to prevent interactions between reflectin and the glass surface. Glass slides and coverslips were sonicated in near boiling 1% Hellmanex cleaning solution for 15 min. After thorough rinsing with Milli-Q water, glass slides were dried with CO_2_ and stored with Drierite desiccant. Coverslips were immersed in 0.1 M NaOH for 15 min, thoroughly rinsed with Milli-Q water, and then dried with CO_2_. They were then heated to 100 °C in closed glass Petri dishes containing Drierite for 10 min, after which 20 μl of 1% methoxy-PEG silane was pipetted onto each 18 × 18 mm coverslip and incubated for 20 min at 100° C. After allowing to cool, coverslips were thoroughly rinsed, sonicated in Milli-Q water for 10 min, and rinsed again. After drying with CO_2_, glass chamber microscope slides were constructed using two strips of Scotch double sided tape to secure the coverslip against the slide.

### FRAP experiments and analysis

One hundred micromolars reflectin A1 containing 5% C199-fluorescein was diluted into freshly 0.22 μm-filtered buffers and then quickly pipetted into PEG-passivated chamber slides which were sealed with fast-drying clear nail polish. Using the FRAP module in a Leica SP8 scanning confocal microscope, a 1 μm diameter circular ROI (region of interest) (“point ROI” in LASX (https://www.leica-microsystems.com/products/microscope-software/p/leica-las-x-ls/) software) was selectively photobleached at 45 to 50% laser power for 150 to 200 ms. The bleached ROI was monitored for 10 s before bleaching and for 25 m post bleaching. For data analysis, videos were stabilized in ImageJ (https://imagej.net/ij/) using the StackReg plugin (https://bigwww.epfl.ch/thevenaz/stackreg/) and the fluorescence intensity of the bleached region, an unbleached region in the droplet, and background were used to correct for photobleaching:

$$F_{corrected}\left(t\right)=\frac{F_{raw}-F_{background}}{F_{nonbleached}-F_{background}}$$

then fully normalized using the equation:

$$I_{norm}=\frac{{\left(I_{bleach}\right)}_{t}-{\left(I_{bleach}\right)}_{0}}{{\left(I_{bleach}\right)}_{pre}-{\left(I_{bleach}\right)}_{0}}$$

where (I_*bleach*_)_*t*_ is the intensity of the photobleached region at time t (I_*bleach*_)_0_ is the intensity of the photobleached region at the time of photobleaching (I_*bleach*_)_*pre*_ is the intensity of the photobleached region prior to photobleaching. To determine the characteristic relaxation time *τ*, data was fit to the equation:

$$y=A_{1}\left(1-e^{-\tau 1t}\right)+B_{1}\left(1-e^{-\tau 2t}\right)$$

using the nonlinear regression function in Mathematica.

### Droplet fusion analysis

Reflectin protein and slides were prepared as described for FRAP and were imaged immediately after chamber slide was prepared. Only fusion events of equally sized droplets were analyzed. Using ImageJ software, the threshold function was used to create binary videos. Aspect ratios were calculated by using the “Fit Ellipse” measurement in “Analyze Particles.” Using the nonlinear regression function in Wolfram Mathematica, the change in aspect ratio over time was fit to the equation:

$$A.R.=1+\left({A.R.}_{0}-1\right)\cdot \;\exp\left(-t/\tau \right)$$

where A.R._0_ is the initial aspect ratio, t is the time, and *τ* is the characteristic relaxation time.

## Data availability

Data are available to be shared upon request. Please contact Reid Gordon at reidwgordon@gmail.com.

## Supporting information

This article contains supporting Information.

## Conflict of interest

The authors declare that they have no conflicts of interest with the contents of this article.
