# Supplementary material for: Charge screening and hydrophobicity drive progressive assembly and liquid–liquid phase separation of reflectin protein
Source: J Biol Chem. 2025 Feb 6;301(3):108277. doi: 10.1016/j.jbc.2025.108277 (PMC11927725; doi:10.1016/j.jbc.2025.108277)
Supplement: Supplementary data [file mmc1.docx]

**Supplemental**


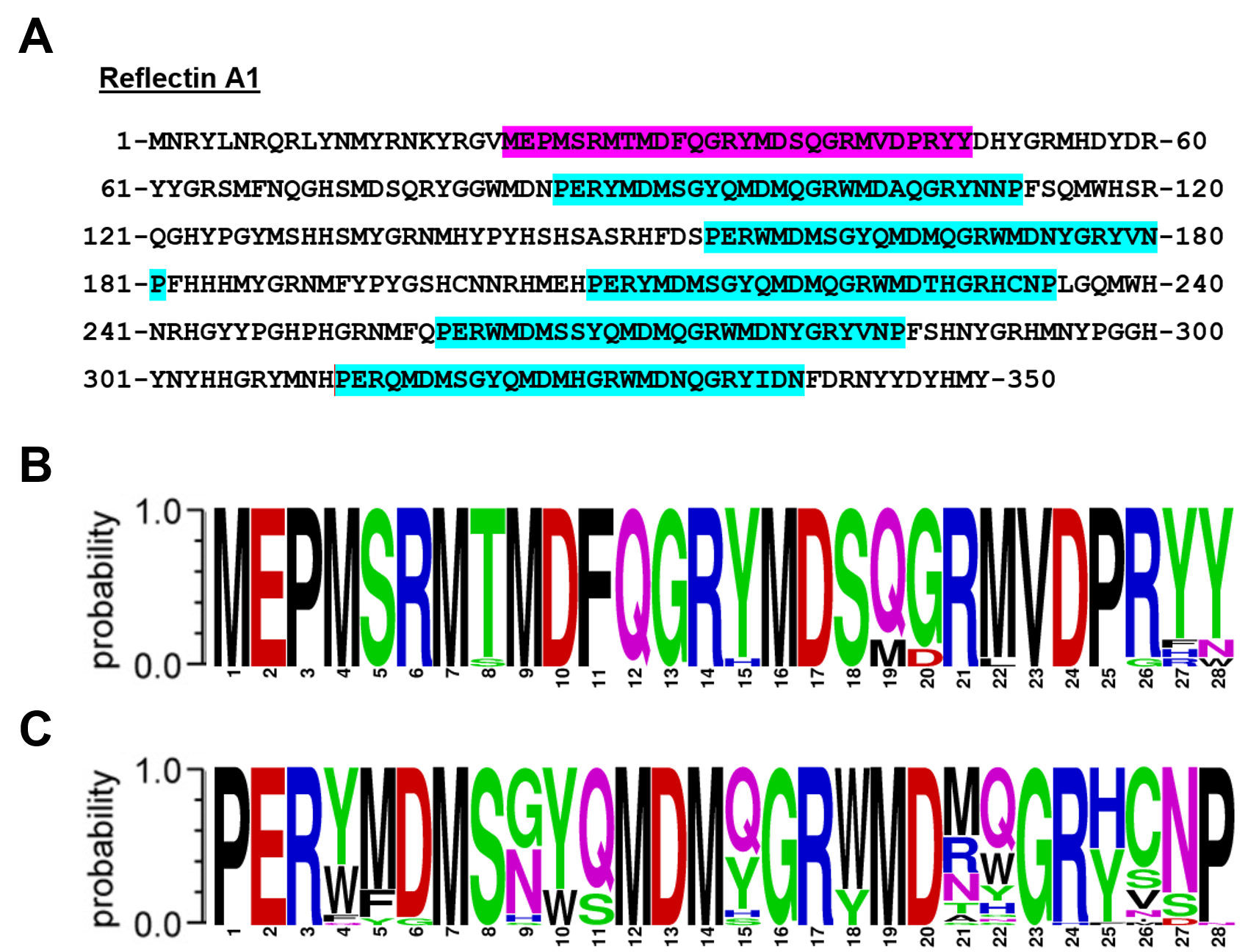


**Figure S1.** A) Amino acid sequence of reflectin A1 from *Doryteuthis opalescens* with N-terminal repeat motif (magenta) and reflectin repeat motifs (cyan). Sequence logo from alignment of the (A) N-terminal repeat motif and (B) reflectin repeat motif from 51 reflectin proteins found in *Octopus bimaculoides, Euprymna scolopes, Sepia oficinialis, Doryteuthis opalescens and Doryteuthis pealeii*.


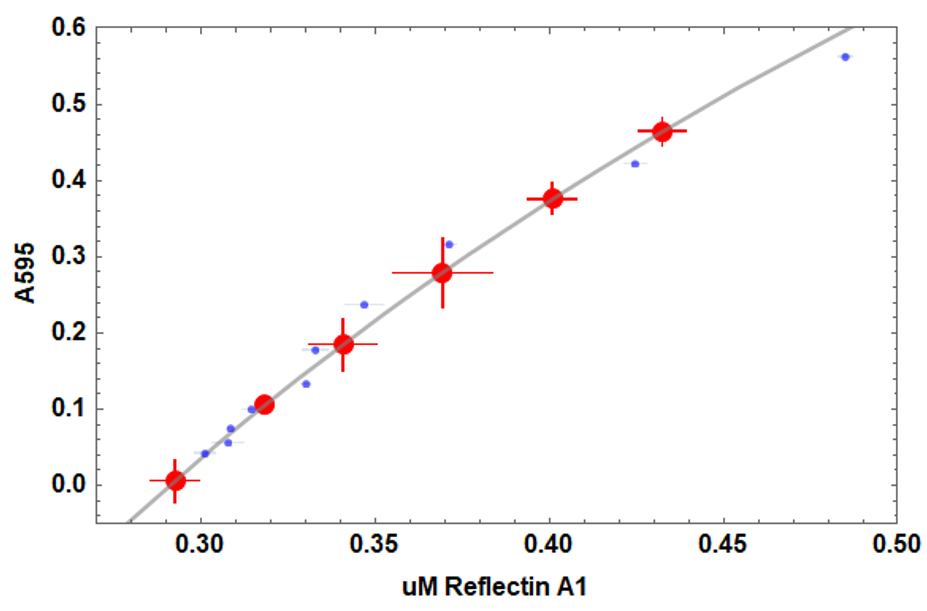


**Figure S2.** Bradford assay used to obtain values shown in Figure 1. Experimentally determined reflectin A1 concentrations using line fit (grey) to protein standards (blue). X-axis is protein concentration and Y axis is absorbance at 595 nm.


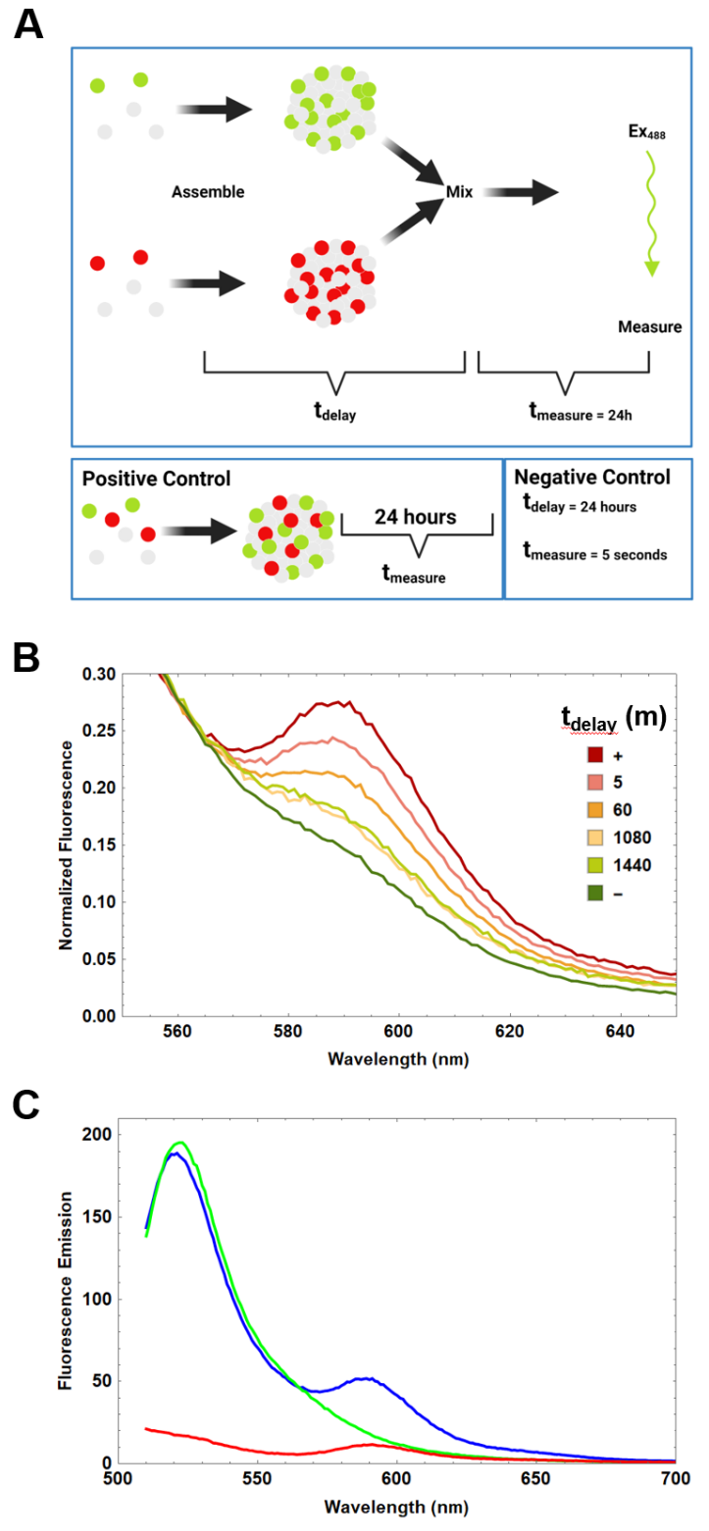


**Figure S3.** A) Design of FRET exchange experiment. Mixtures of reflectin A1 containing either 5% fluorescein-labeled single cysteine mutant C232S (A1 C199-F) or 5% rhodamine sulfate-labeled C232S (A1 C199-R) were separately then assembled by dilution into pH 7 25 mM MOPS buffer. After t_delay_ assemblies containing 5% A1 C199-F were mixed with those containing 5% A1 C199-R and incubated 24 hours at 20° C. Samples were excited with 488 nm light corresponding to the absorption spectra of donor fluorophore fluorescein and emission spectra from 510-700 nm recorded. A mixture containing both labels was driven to assembly as the positive control. For the negative control t_delay_ was 24 hours and t_measure_ was 5 seconds. B) Spectra normalized to 520 nm (fluorescein emission maximum). C) Raw fluorescence emission of reflectin A1 assemblies labeled with 5% fluorescein (green), 5% rhodamine sulfate (red) and the positive control containing both fluorescent labels (blue) excited at 488nm.


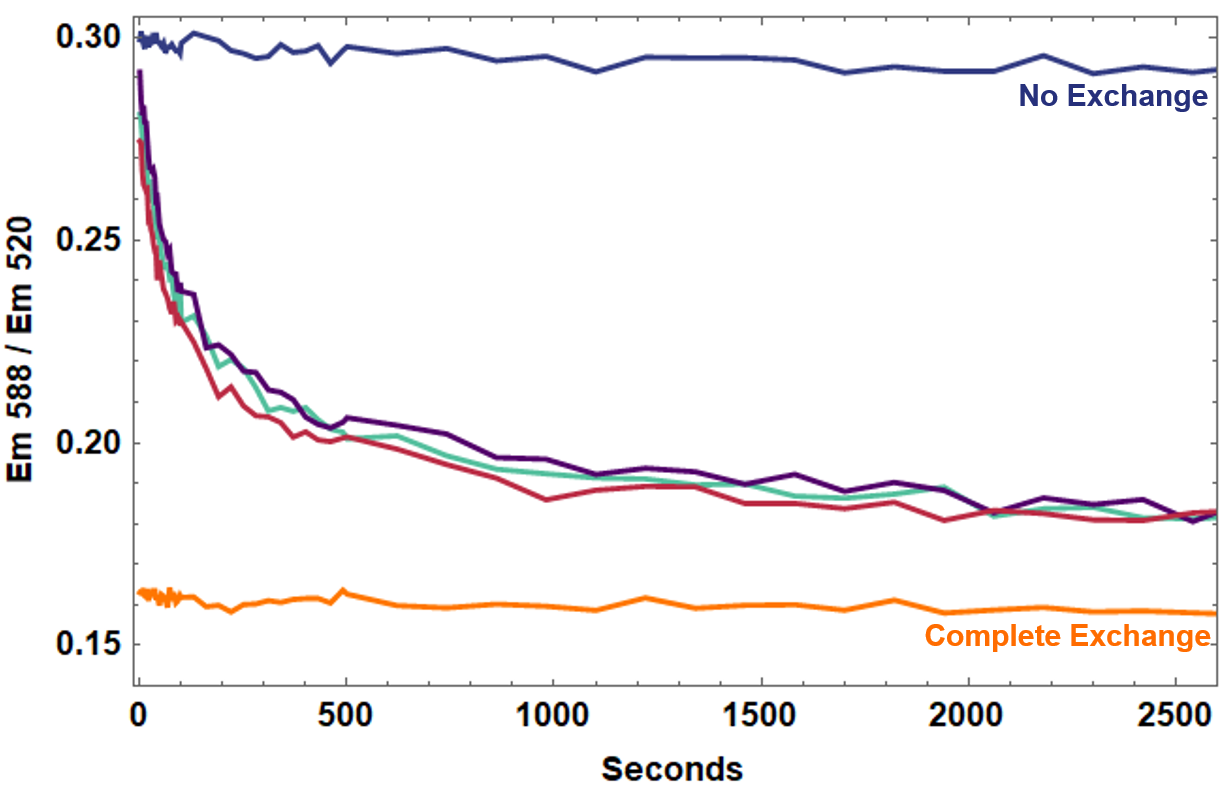


**Figure S4.** Change in FRET signal as a function of time. Experimental conditions and controls are identical to those for **Figure 2**, except labeled assemblies were diluted 1:5 with unlabeled assemblies. Three experimental repeats are shown. The top blue line is the negative control and represents no exchange of particles between reflectin assemblies. The bottom orange line is the positive control representing complete dynamic exchange of particles between reflectin assemblies.


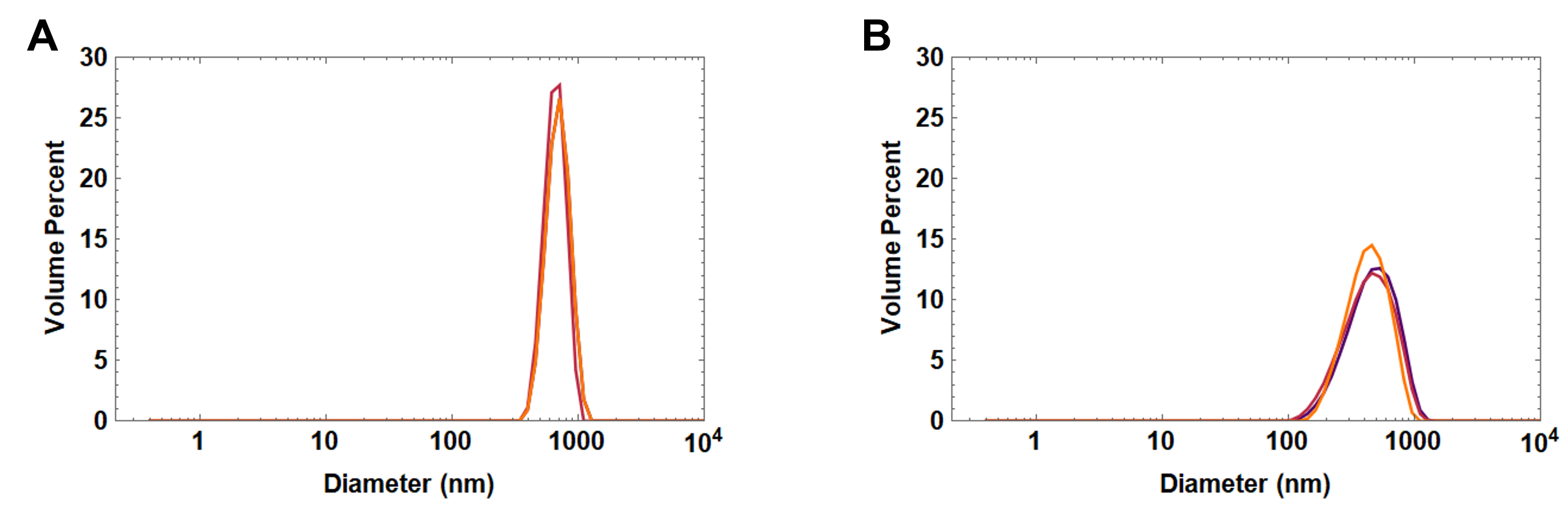


**Figure S5.** DLS size distributions of fluorescently labeled reflectin A1 C232S. A) Size distribution by volume percent of A) C199-R (rhodamine labeled reflectin A1 C232S) and B) C199-F (fluorescein labeled reflectin A1 C232S).


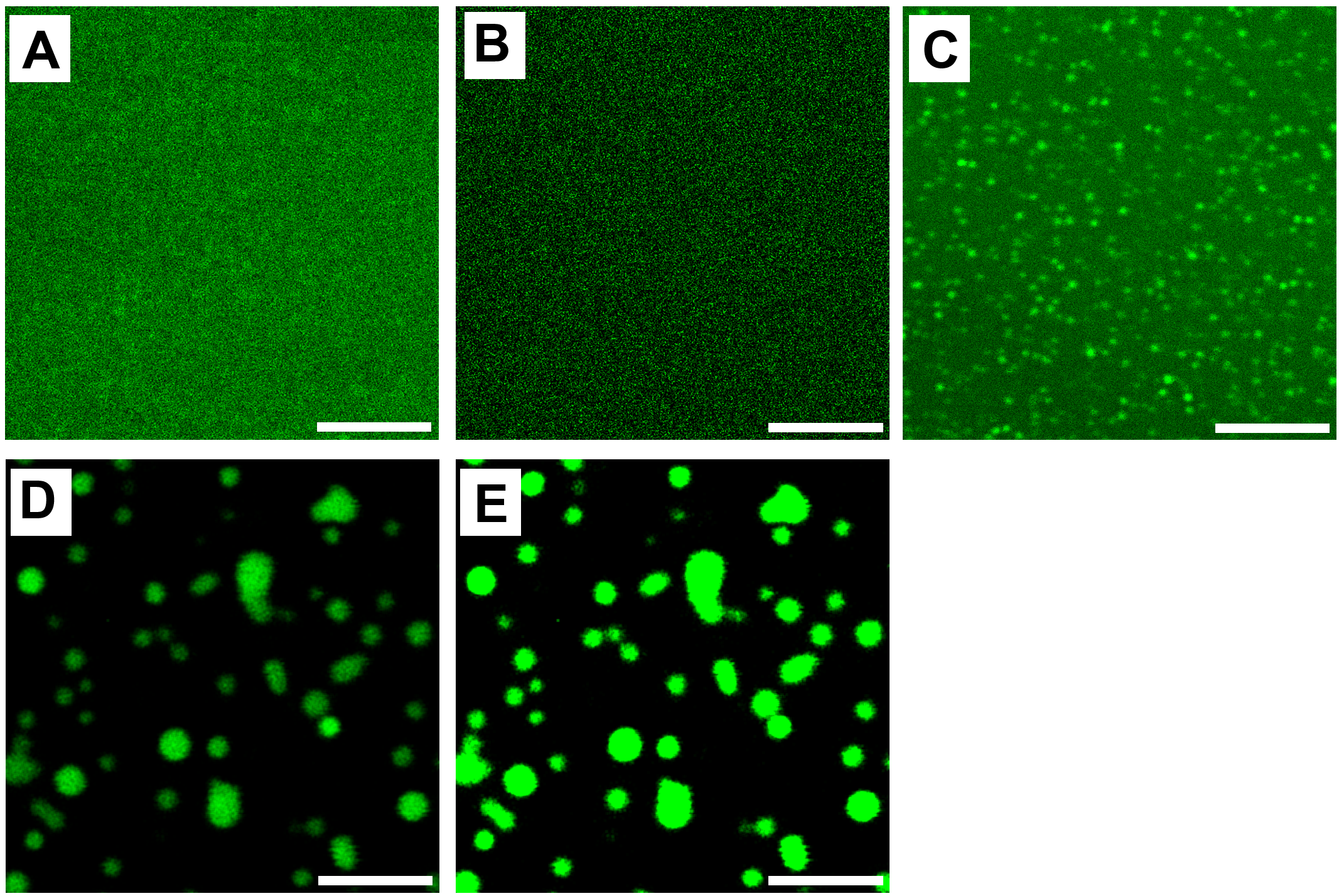


**Figure S6.** Reflectin A1 assembly sizes increase until strongly partitioned liquid droplets are formed. 100 μM reflectin A1 containing 5% A1-C199F diluted into increasing concentrations of NaCl in acetic acid buffer (pH 4 25 mM) until the liquid phase boundary is crossed. A) At 80 mM NaCl no light-resolvable structures are detected but assemblies are present as determined by DLS. B) Same as (A) but imaged using an mPEG-passivated coverslip. C) At 90 mM NaCl ca. 500 nm d. particles are present. D) At 100 mM NaCl surface-wetting liquid droplets are present. E) Same image as C but at the same exposure values as (A,B) for comparison of protein partitioning into dense phase. Scale bars = 5 µm.


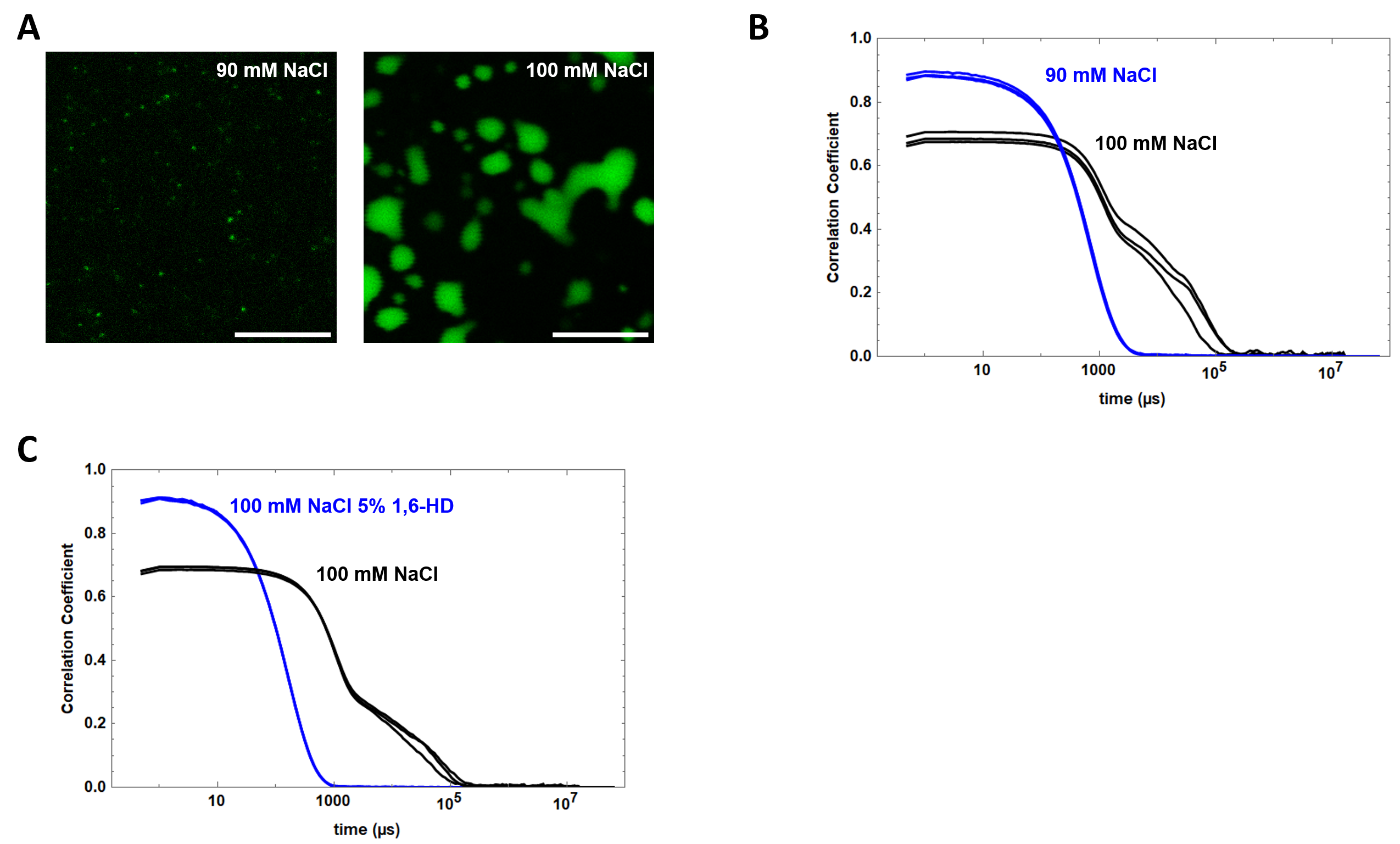


**Figure S7.** Confocal microscopy and DLS of 4 μM reflectin A1 in acetic acid buffer (pH 4, 25 mM) diluted into respective buffers. A) Confocal microscopy and (B) autocorrelation functions of reflectin A1 in 90 mM NaCl and 100 mM NaCl. C) Autocorrelation functions of reflectin A1 in 100 mM NaCl and in the same solution containing 5% 1,6-hexanediol. Autocorrelation functions are 3 successive measurement replicates of each experimental condition. Scale bars = 10 µm.


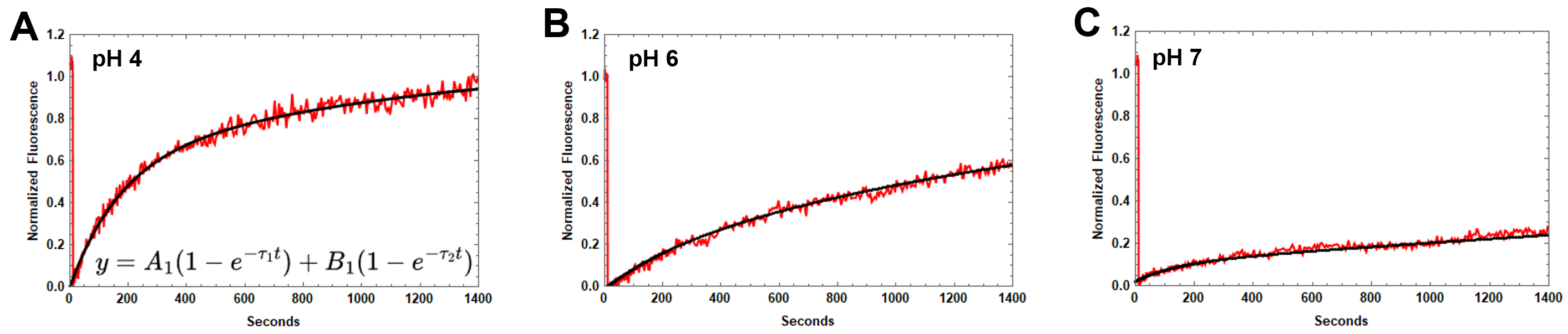


**Figure S8.** Examples of fits of individual FRAP experiments to an equation of exponential decay for (A) pH 4, (B) pH 6 and (C) pH 7.


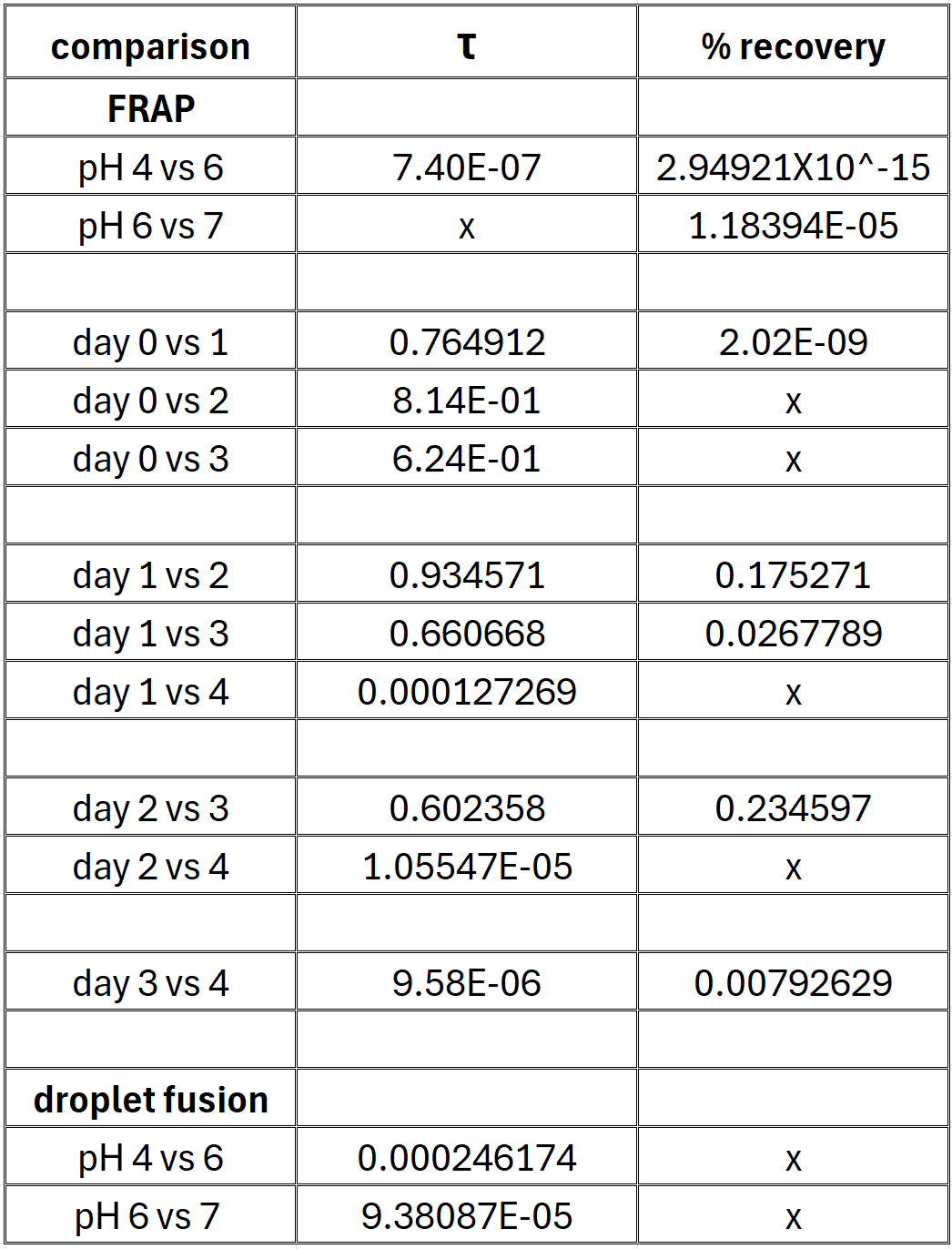


**Figure S9.** P-values obtained from statistical testing of FRAP and droplet fusion experiments. Statistical significance was determined by one-way ANOVA testing. ‘X’ denotes comparisons that were not made.


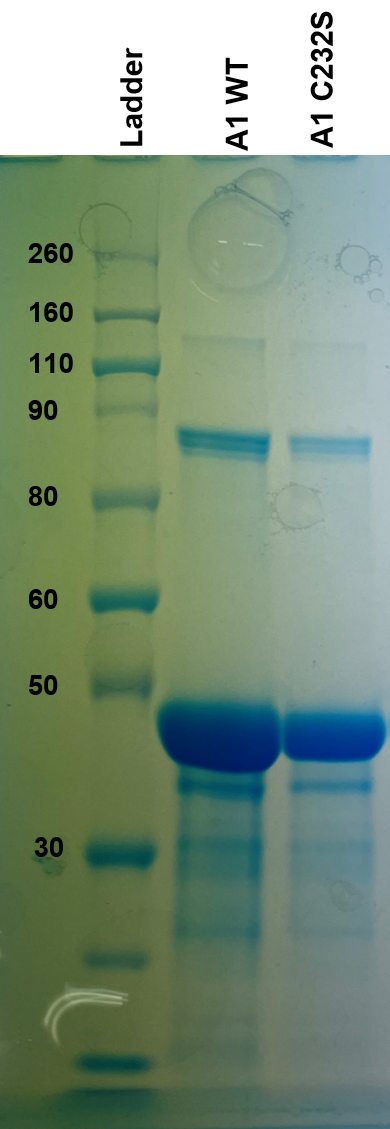


**Figure S10.** SDS-PAGE gel of resolubilized reflectin A1 and A1 C232S mutant.
